# Liver fibrosis progression and clinical outcomes are intertwined: role of CD4+ T-cell count and NRTI exposure from a large cohort of HIV/HCV-coinfected patients with detectable HCV-RNA: A MASTER cohort study

**DOI:** 10.1097/MD.0000000000004091

**Published:** 2016-07-22

**Authors:** Emanuele Focà, Massimiliano Fabbiani, Mattia Prosperi, Eugenia Quiros Roldan, Francesco Castelli, Franco Maggiolo, Elisa Di Filippo, Simona Di Giambenedetto, Roberta Gagliardini, Annalisa Saracino, Massimo Di Pietro, Andrea Gori, Laura Sighinolfi, Angelo Pan, Maria Concetta Postorino, Carlo Torti

**Affiliations:** a University Department of Infectious and Tropical Diseases, University of Brescia and Brescia *Spedali Civili* General Hospital, Brescia; b Clinic of Infectious Diseases, “*San Gerardo de’ Tintori*” Hospital, Monza, Italy; c Department of Epidemiology, College of Public Health and Health Professions & College of Medicine, University of Florida, Gainesville, FL; d Clinic of Infectious Diseases of *“Papa Giovanni XXIII*” Hospital, Bergamo; e Institute of Clinical Infectious Diseases of Catholic University of Sacred Heart, Rome; f Clinic of Infectious Diseases, University of Bari, Bari; g Clinic of Infectious Diseases of “*Azienda Ospedaliera SM. Annunziata*”, Firenze; h Clinic of Infectious Diseases of “*Azienda Ospedaliera S. Anna*”, Ferrara; i Clinic of Infectious Diseases of “*Istituti Ospitalieri*”, Cremona; j Infectious Diseases Unit, University “*Magna Graecia*”, Catanzaro, Italy.

**Keywords:** antiretroviral therapy, clinical events, FIB-4, HCV, HIV, liver fibrosis

## Abstract

**Introduction:**

Patients coinfected with human immunodeficiency virus (HIV) and hepatitis C virus (HCV) suffer from faster progression of liver fibrosis (LF) and have greater risk of worse clinical outcomes. We evaluated predictors and incidence of these events in a large multicentre cohort.

**Methods:**

We selected all HIV-infected patients starting a first-line combination antiretroviral therapy (cART), with detectable HCV-RNA, without exposure to interferon/ribavirin, with ≥2 fibrosis-4 index (FIB-4) classifications before cART. Kaplan–Meier analysis was used to estimate incidence of clinical events (AIDS, non-AIDS related, deaths) and LF progression (via transitions: from FIB-4 class 1 to 2 or 3, from class 2 to class 3, and worsening by 0.5 point). Multivariate Cox regression was used to assess predictors, baseline, or time updated.

**Results:**

One thousand four hundred thirty-three patients were selected. Overall, 745 clinical events occurred, with an incidence of 7.6% over 9811 person-year of follow-up (PYFU) and a median survival time of 9.36 years. Incidence of LF progression from FIB-4 class 1 to 2 or 3 was 12.4%, and from FIB-4 class 2 to 3 was 7% with a median survival time of 5.67 and 10.35 years, respectively. At multivariate analyses, intravenous drug use and time-updated gamma-glutamyl transferase (γGT) were negative predictors for any outcomes, either clinical or FIB-4 progression. Higher CD4+ T-cell protected from clinical events, and lower HIV-RNA and higher CD4+ T-cell appeared to protect from FIB-4 transitions. Moreover, independently from the viro-immunological status, current FIB-4 class 3 predicted clinical events. Occurrence of AIDS and cardiovascular/kidney events were significant predictors of 0.5 point worsening and transitions of FIB-4, respectively. Prolonged exposure to nucleos(t)ide reverse transcriptase inhibitors (NRTI) was a negative predictor for any outcomes.

**Conclusion:**

Both clinical and LF progression in HIV/HCV-coinfected patients depend strongly on immune status. Intravenous drug users and patients with high γGT (a possible proxy for alcohol abuse) are most-at-risk for both outcomes, as well those who had prolonged exposures to the NRTI class. Therefore, these patients should be prioritized for the access to anti-HCV therapy and a test-and-treat strategy should be implemented for early initiation of cART. Possible benefits of NRTI sparing regimens in HIV/HCV-coinfected patients should be investigated.

## Abstract 2

Supplemental Digital Content is available in the text

## 1 Introduction

In human immunodeficiency virus (HIV)-infected patients, chronic hepatitis C virus (HCV) infection is common due to shared modes of transmission, and it is characterized by an accelerated progression of liver fibrosis (LF).^[1,2]^ This can potentially lead to cirrhosis, hepatocellular carcinoma and decompensated liver disease, accounting for a substantial morbidity and mortality in this population even in the combination antiretroviral therapy (cART) era.^[3–5]^ Consequently, all HIV-infected patients with HCV coinfection should be treated for HCV, but in many countries, including Italy, only those with a significant degree of LF or comorbidities are prioritized due to cost constraints.^[6]^

Staging of LF and evaluation of LF progression are crucial to providing prognostic information and guiding physicians in deciding priority and timing to start anti-HCV treatment.^[7]^ Liver biopsy is considered the gold standard for staging of LF.^[7]^ However, it is an invasive procedure and has limited patient acceptability.^[8]^ Moreover, results are affected by sampling errors and inter/intraobserver variability,^[9,10]^ and its use is unpractical to assess the progression of LF over time, as biopsies would be required repeatedly. For these reasons, several methods for noninvasive assessment of LF have been proposed.^[11,12]^ Some of these methods, such as the fibrosis-4 (FIB-4) index, are based on serum biomarkers, reflect with acceptable accuracy the degree of LF and are significant predictors of hepatic decompensation and liver-related deaths both in HIV mono-infected and in those with positive HCV antibodies.^[13–15]^

However, comprehensive prognostic models for morbidity and mortality in HIV-infected patients coinfected with HCV are lacking or imperfect. First, it has not been assessed if, and to what extent, HIV by itself and HIV-related complications (such as AIDS and not AIDS events) impact LF progression, which in turn may influence the risk of mortality. This hypothesis is based on a previous study of our group in HIV-infected patients not coinfected by HCV.^[13]^ Indeed, HIV RNA control induced by cART appeared to counteract LF progression, an observation that has been confirmed using different methods (e.g., transient elastometry) in a patient group mainly composed by HIV mono-infected individuals.^[16]^ Therefore, HIV by itself may have a direct effect on fibrogenesis, and variables related with the HIV natural history (such as HIV RNA, CD4+ T-cell count, and AIDS events) may have a significant effect in accelerating or predicting LF progression in HIV/HCV-coinfected patients. Second, an important limitation of the current prognostic models is that patients with positive anti-HCV antibodies (HCV-Ab) are selected, without consideration of HCV-RNA (in some cases, patients have spontaneously eradicated HCV), and this may have confounded risk estimates associated with other variables tested.^[15]^ In conclusion, there is a need for more studies conducted in HIV-infected patients with confirmed HCV coinfections (assessed through positive HCV-RNA) to explore whether FIB-4 plays a role as a predictor of clinical evolution and survival in relationship with HIV-related variables and occurrence of AIDS and non-AIDS-related events.

For the reasons above, we have designed a retrospective, longitudinal study to evaluate incidence and predictors of LF progression, as well as of clinical events and death in a large cohort of HIV/HCV-coinfected patients with detectable HCV-RNA and developed clinically comprehensive models for predicting both LF progression and death in these patients.

## 2 Methods

### 2.1 Selection of patients

The study was conducted within the MASTER (*MAnagement Standardizzato di TErapia antiRetrovirale*, Standardized Management of Antiretroviral Therapy) cohort, a longitudinal multicenter cohort consisting of a general HIV-infected patient population in nine referral centers throughout Italy (http://www.mastercohort.it).^[17]^ The distinguishing characteristic of this cohort is that real-life data are recorded in a shared electronic chart (Health & Notes 3.5W, Healthware S.p.A., Naples, Italy) used in the participating centers. Data are recorded over a standardized time scale every 3/6 months, with merging and data cleaning performed at the coordinating center every 6 months.^[17]^ The electronic database is used to manage everyday activity of the outpatient HIV clinics in each center. The resulting cohort is therefore an open cohort in which patients are enrolled without preselection. Subjects gave written informed consent for participation in the observational cohort, and each site obtained approval by its ethics committee.

Within this cohort, we performed a retrospective study of HIV-infected patients with HCV coinfection, defined as positive HCV-Ab and detectable HCV-RNA. Patients aged 18 years or more, naïve to antiretroviral therapy at the time of enrollment in the cohort (from January 1989 to December 2014), with at least 2 available FIB-4 results obtained 3 months apart prior to starting cART were included. Exclusion criteria were as follows: HBsAg positivity, reported actual or past alcohol abuse, previous anti-HCV treatment with interferon ± ribavirin, diagnosis of cirrhosis or hepatocellular carcinoma (HCC) and baseline advanced LF according to FIB-4 index (baseline FIB-4 class 3, see below).

### 2.2 Assessment of liver fibrosis

LF was estimated using FIB-4 score, calculated using Sterling formula,^[13]^ as follows: age [years] × AST [IU/L]/platelet count [expressed as platelets × 10^9^/L] ×  (ALT^1/2^[IU/L]). As previously described,^[16]^ LF was ranked using standard cut-off values into 3 classes corresponding to increased severity of LF: FIB-4 class 1, ≤1.45 (no clinically significant fibrosis); FIB-4 class 2, from 1.46 through 3.25 (moderate fibrosis); FIB-4 class 3, >3.25 (significant fibrosis or cirrhosis).

### 2.3 Follow-up and study outcomes

Patients were followed from baseline (date of first available FIB-4 result confirmed in the same class at least 3 months apart) to the time of death, outcome occurrence (see below) or to the last available visit. Follow-up was also interrupted in the case of incident HBsAg positivity, diagnosis of HCC, prescription of anti-HCV treatment or spontaneous HCV-RNA clearance, and when patient were lost to study follow-up.

The 4 outcomes of the study were as follows: composite outcome, defined as death or the occurrence of at least one of the following clinical events, liver-related disease, cardiovascular event, kidney-related disease, neurological event, or AIDS-defining event, classified as presented in a recent MASTER cohort study^[18]^; transition from baseline FIB-4 class to any higher class during the follow-up period (i.e., from class 1 to class 2 or 3; or from class 2 to class 3); transition from baseline FIB-4 class 1 or 2 to FIB-4 class 3 during the follow-up period; increase of FIB-4 score more than half a point from baseline.

Owing to the fact that these scores may fluctuate or revert back to lower classes after initial worsening, FIB-4 class transition was defined in our analyses as a transition to higher classes confirmed in 2 subsequent measurements at least 3 months apart (unless follow-up was truncated). Right censoring of follow-up times was employed for all outcomes, namely end of follow-up for outcome 1, and end of follow-up, death, HCC, start of interferon therapy, HCV-RNA clearance.

### 2.4 Data collection

Baseline data included date of birth, gender, ethnicity, date of first HIV diagnosis, risk factors for HIV acquisition, CDC stage, nadir CD4+ T cell count. The following parameters were collected at baseline and every 3 to 6 months: antiretroviral regimens prescribed (categorized as: naïve; ritonavir boosted protease inhibitors [PI/r]-based; protease inhibitors [PI]-based; nucleoside reverse transcriptase inhibitors [NRTI] or nonnucleoside reverse transcriptase inhibitors [NNRTI]-based regimens, or other regimens; treatment interruption), CD4+ T-cell and CD8+ T-cell counts, HIV-RNA, AST, ALT, total bilirubin, γ-glutamyl transferase (γGT) level (as a surrogate marker of alcohol intake, even not reported by the patient)^[19]^ and platelet count. FIB-4 index was calculated at baseline and during follow-up using data collected at each time point.

### 2.5 Statistical analysis

Continuous variables were described by median and interquartile range (IQR), while categorical variables were described by frequencies. Study outcomes were evaluated in terms of incidence and prevalence with Poisson confidence intervals. Survival probability estimates at various time points, as well as crude hazards were assessed via Kaplan–Meier analysis. Multivariate survival analysis was performed using Cox regression for all outcomes, with the inclusion of time-updated covariates.

A 2-tailed *P*-value of <0.05 was considered statistically significant. All analyses were performed using the R language for statistical computing (https://www.r-project.org/).

## 3 Results

### 3.1 Population characteristics

A total of 1433 HIV positive patients coinfected with HCV fulfilled the inclusion criteria and were included in the analysis. Baseline population characteristics are summarized in Table 1. Most patients were males, predominantly young (median age was 34 years) and acquired HIV infection through intravenous drug use. According to FIB-4, baseline LF was classified as class 1 or 2 in 70.41% and 29.59% of patients, respectively.

**Table 1:**
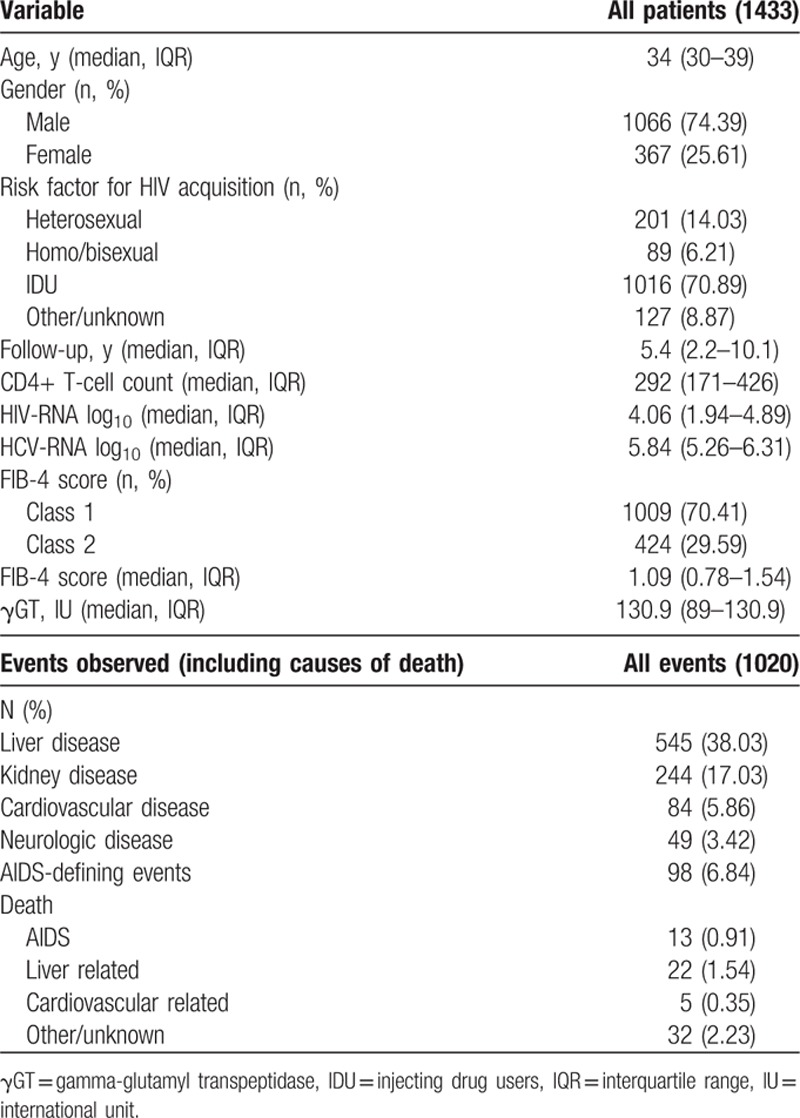
Baseline characteristics of patients.

### 3.2 Incidence of clinical outcomes and liver fibrosis progression according to FIB-4

**Figure 1:**
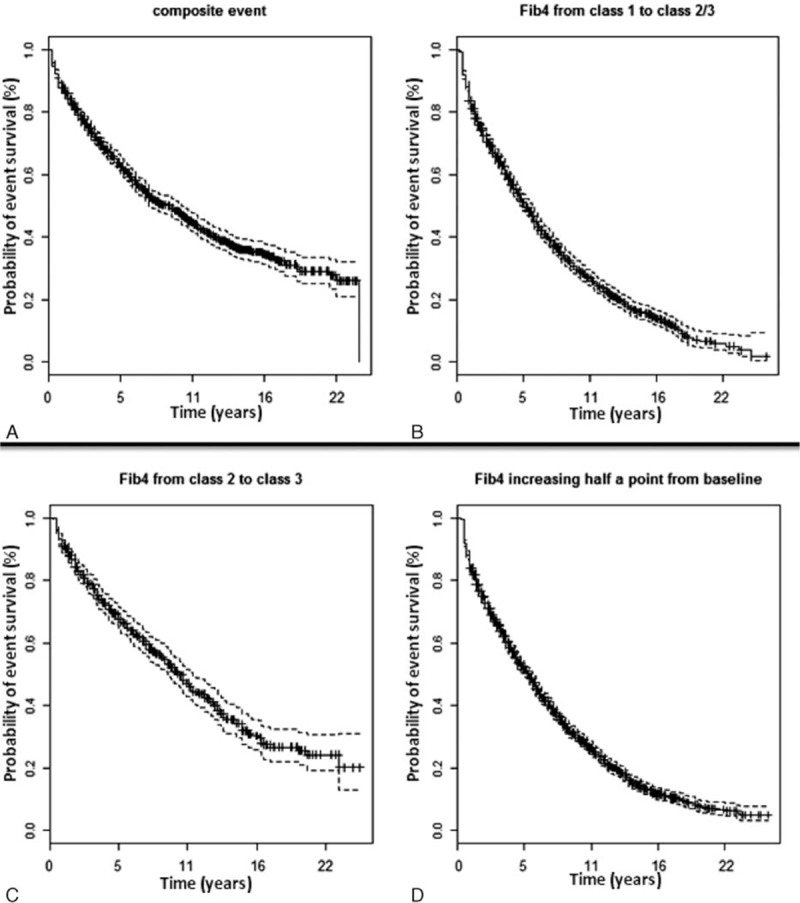
Incidence of clinical outcome and liver fibrosis progression according to FIB-4: (A) composite event (clinical events and death); (B) FIB-4 increase from class 1 to class 2/3; (C) FIB-4 increase from class 2 to 3; (D) FIB-4 increase of half a point from baseline.

A total of 745 clinical events or deaths occurred overall (Fig. 1A), with an incidence of 7.6% (95% confidence interval [CI]: 7.1%–8.2%) over 9811 PYFU and a median survival of 9.36 (95% CI: 7.64–10.1) years. A total of 525 liver-related events occurred in patients without these events at baseline (with an incidence of 4% over 11,671 PYFU and a median survival of 18.73 years). All the events contributing to the composite outcome including causes of death are shown in Table 1. The composite outcome counts only the first qualifying event if more than 1 happen over time (745 over a total of 1020); on the liver event outcome, liver events were not considered after occurrence of censoring conditions (HCC/interferon/HCV clearance).

As shown in Fig. 1B, incidence of LF progression from FIB-4 class 1 to class 2 or 3 was 12.4% (95% CI: 11.7–13.2), accounting for 1107 events with a median survival time of 5.67 years (95% CI: 5.17–6.16) over 8913 PYFU. Incidence of LF progression from FIB-4 class 2 to 3 (Fig. 1C) was 7% (95% CI: 6.3–7.8); we recorded 365 events over 5191 PYFU with a median survival time of 10.35 years (95% CI: 9.36–11.33). Lastly, FIB-4 increase of at least half a point from baseline was observed in 1644 cases with an incidence of 12.6% (95% CI: 12–13.2) over 13,079 PYFU and an estimated median survival time of 5.91 years (95% CI: 5.42–6.16) (see Fig. 1D).

### 3.3 Predictors of death, clinical, and liver fibrosis progression according to FIB-4

**Figure 2:**
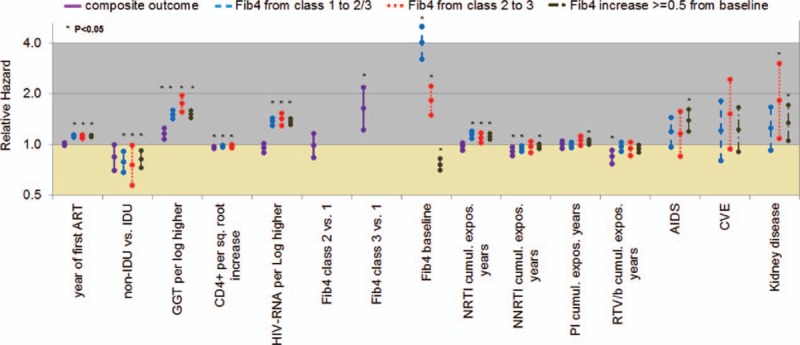
Predictors of death, clinical events, and liver fibrosis progression according to FIB-4. Adjusted for: gender, increased HCV-RNA (per Log higher), other antiretrovirals cumulative exposure years, neurological events. Variables with *P* < 0.1 for at least 1 outcome are shown. ART = antiretroviral therapy, IDU = injecting drug users, GGT = gamma-glutamyl transferase, NRTI = nucleos(t)ide reverse transcriptase inhibitors, NNRTI = nonnucleoside reverse transcriptase inhibitors, PI = protease inhibitors, RTV/b = ritonavir boosted protease inhibitors, CVE = cardiovascular events.

Predictors of the 4 outcomes were investigated using multivariate Cox regression analysis with both time-fixed (collected at baseline) and time-updated (collected at baseline and during follow up) variables (detailed results of the multivariable analysis are reported in the Supplementary Material and depicted in Fig. 2).

At multivariate analysis, intravenous drug use (as a risk factor for HIV acquisition) and current γGT increase (as time-dependent variable) were significant risk factors for any outcomes. Indeed, relative hazard ratios (HR) ranged from 0.76 (95% CI: 0.58–1; *P* = 0.0460) for intravenous drug use for transition of FIB-4 from class 2 to class 3 to 0.84 (95% CI: 0.70–1; *P* = 0.05) for intravenous drug use for the composite clinical outcome. Also, an increased current γGT (per Log higher) increased the risk of composite clinical events (HR 1.16, 95% CI: 1.08–1.26; *P* = 0.0001) and the risk of transition of FIB-4 from class 2 to class 3 (HR 1.76, 95% CI: 1.57–1.96; *P* < 0.0001). Higher CD4+ T-cell count during follow-up predicted a lower risk of clinical events and deaths (composite outcome), while both lower HIV-RNA and higher CD4+ T-cell count appeared to protect from FIB-4 transitions (both for progression to class 2 or 3 and for the ≥0.5 point increase). For example, with respect to FIB-4 transition from class 2 to class 3, increased HIV-RNA (per Log higher) accounted for a HR of 1.42 (95% CI: 1.30–1.54; *P* < 0.0001).

Occurrence of AIDS-defining events predicted ≥0.5 point increase in FIB-4 (HR 1.39, 95% CI: 1.19–1.61; *P* < 0.0001), while kidney events were significant risk factors for both transition from class 2 to class 3 (HR 1.82, 95% CI: 1.09–3.03; *P* = 0.0221) and ≥0.5 point increase in FIB-4 (HR 1.35, 95% CI: 1.06–1.72; *P* = 0.0156). Conversely, we observed that FIB-4 class 3 (time updated) was a predictor of the composite outcome (any clinical events and death), independently from the other variables tested (HR 1.64 vs class 1, 95% CI: 1.23–2.19; *P* = 0.0008). Lastly, we found that time-updated FIB-4 was a significant predictor of liver-related events independently from other factors (hazard ratio, HR: 1.33, 95% CI: 1.10–1.62; *P* = 0.0038 for FIB-4 class 2 vs 1, and HR: 2.40, 95% CI: 1.75–3.30; *P* < 0.0001 for FIB-4 class 3 vs 1).

From analyzing the impact of antiretroviral therapy on LF progression, a prolonged exposure to NRTIs appeared to be a consistent predictor for FIB-4 evolution. For example, prolonging exposure by one year accounted for a HR of 1.10 (95% CI: 1.03–1.18; *P* = 0.0077) for FIB-4 transition from class 2 to class 3. For other drug classes, results were less consistent, with NNRTI protecting from transition to class 2 or 3, and use of single PI (but not boosted by ritonavir) promoting ≥0.5 point increase of FIB-4. Use of NNRTI and of PI/r (but not the use of unboosted PI) displayed a protective effect for the composite clinical outcome: for NNRTI, the HR was 0.92 (95% CI: 0.86–0.97; *P* = 0.0043) and for boosted PI regimens the HR was 0.85 (95% CI: 0.77–0.93; *P* = 0.0004).

## 4 Discussion

This study evaluated incidence and predictors of LF progression (estimated by FIB-4 index) and outcomes of HIV/HCV-coinfected patients included in the Italian MASTER cohort. We feel that our findings are innovative and applicable to clinical practice.

Most importantly, we found that incidence rate of LF progression in HIV/HCV-coinfected patients is far higher than that estimated in HIV mono-infected patients belonging to the same cohort.^[13]^ For instance, the incidence of transition to FIB-4 >3.25 was 0.013 per PYFU in our previous analysis^[13]^ vs 0.070 per PYFU in the present study. Therefore, although patients seem to be protected by cART especially in the first years after its prescription (as recently suggested by prospective data in a small patient cohort),^[20]^ LF progressed faster afterwards, making HIV/HCV-coinfected patients a population that should be prioritized for access to HCV treatment. Unfortunately, the high cost of the new directly acting antivirals limits the applicability of such therapy in many countries, such as in Italy. For this reason, it is still important to define the best predictors of LF progression to address drug prescription to the most-at-risk patients.

A strong association between higher baseline FIB-4 and LF progression was observed. This may indicate that patients with higher degrees of LF should be considered a high priority group for anti-HCV therapy. Also, we were able to identify other predictors of LF progression, which may help to individualize drug prescription and intensity of clinical monitoring.

Current or past intravenous drug use appeared to predict LF progression and death or clinical events (composite outcome). So, this group of patients should be strictly monitored for LF progression and anti-HCV treatment should be proposed earlier if not contraindicated.

As in HIV mono-infected patients,^[13]^ abnormal γGT levels were associated with faster transition to higher FIB-4 classes, possibly related to undisclosed alcohol consumption. However, other factors could have contributed to this finding such as liver toxicity due to comedications or some antiretroviral drugs. On the other hand, increased levels of γGT predicted death or clinical events at the composite outcome, which is likely due to the severity of liver disease and liver-related complications.

We found that AIDS-defining and kidney-related events were significant predictors of ≥0.5 point worsening in FIB-4, while clinical events (in particular kidney related) increased the risk of transitions to FIB-4 class 3. Clearly, worsening of liver function may accompany or lead to several clinical problems including not only liver-related events, but also neurological, cardiovascular, kidney-related, and AIDS-defining events. The role of these clinical events for prediction of LF progression is somewhat unexpected and not well explained. However, Lapadula^[18]^ previously demonstrated in our cohort that patients who experienced previous AIDS-defining events were more at risk for non-AIDS-defining events, so these factors appear to be intertwined.

Consistent with this hypothesis, better immune recovery appeared to reduce the overall disease progression in HIV/HCV-coinfected patients both in terms of clinical events and LF in the present study. In fact, although only higher CD4+ T-cell count protected from death and composite outcome, both higher CD4+ T-cell count and lower HIV-RNA seemed to protect from worsening of LF. These findings were previously demonstrated both in HIV mono-infected patients and in HCV-coinfected patients.^[15,20–23]^ Our data confirm that viro-immunological parameters may have a role in LF progression. A cross-talk among HIV and HCV proteins in coinfected patients modulates the natural history, the immune responses, and the life cycle of both viruses. These effects are mediated by immune mechanisms and by cross-talking between the 2 viruses which could interfere with host defense mechanisms, as recently reviewed by Liberto.^[24]^ From a clinical point of view, the current data strongly indicate the need for starting both cART and HCV therapy as soon as possible.

A lower risk of LF progression was observed in recent calendar years. This could be ascribed to the shorter follow-up of patients, but also to earlier initiation of antiviral therapy according to the recent guidelines^[25]^ or to the better liver tolerability of new generation of antiretroviral drugs. In fact, in our multivariate analysis, prolonged exposure to NRTIs predicted LF progression. A potential explanation could be the potential liver toxicity due to antiretroviral drugs belonging to this class (particularly dideoxynucleoside analogs). Indeed, these drugs were associated with cryptogenic liver disease and microvesicular steatosis.^[26,27]^ In addition, we found a protective role of both exposure to NNRTIs and to ritonavir-boosted PIs for the composite outcome. Moreover, NNRTI exposure appeared to counteract progression of LF (from class 2 to class 3). These associations could be explained by the overall advantages of these antiretroviral regimens on the clinical outcome compared to suboptimal regimens based on NRTI only.^[28]^ However, the use of these regimens has been abrogated in the modern cART era. Perhaps more important is the apparent protective effect of NNRTI containing regimens, which could be ascribed to better tolerability and metabolic profile of this kind of therapy.^[29]^ We hypothesized that containment of insulin resistance and lipid increase attributed to these regimens may have given some benefits to the liver of our patients in terms of steatosis, slowing down progression of LF. Lastly, exposure to unboosted PIs seemed to promote LF progression (>0.5 point FIB-4 increase). We are not sure whether this is a true cause–effect finding or simply reflects the habit to prescribe these antiretroviral regimens in coinfected patients with more advanced liver disease because of the perception of better tolerability or pharmacokinetic profiles.^[30,31]^ However, this effect was not confirmed for the other FIB-4 endpoints, so its clinical meaningfulness is reduced.

Some limitations should be discussed when interpreting the results of our study. First, LF was monitored using a noninvasive score, which is not the gold standard for LF assessment. However, liver biopsy is impractical for serial evaluations and not applicable in longitudinal cohort studies that require multiple evaluations of LF. Transient elastography could have been an alternative tool,^[32]^ but this technique has become available only in recent years, thus limiting the length of follow-up; moreover, this tool could not have been applied to such a large population for practical reasons, and furthermore, elastography may be affected by operator-based biases. Despite a recent study found that noninvasive markers are not optimal,^[20]^ these markers were already validated for estimating LF^[13]^ and predicted hepatic decompensation and death in cohorts of patients.^[4,15]^ For these reasons, the European Association for the Study of the Liver (EASL) provided specific guidelines for the use of these scores in clinical practice.^[12]^ Second, in dealing with a possible effect of antiretroviral drugs, we did not try to disregard the impact of individual compounds because of the risk of introducing biases when statistical analysis is “forced” to provide such levels of details given the fact that combination of drugs are prescribed. Indeed, we feel that the impact of individual compounds merits to be addressed in specific studies, either in patients drug-naïve on treatment or after switching.

The innovative strength of this study, making it one of the few in current literature in this field, was that we did not include all HCV-Ab positive patients but only those with detectable HCV-RNA (i.e., actual HIV/HCV coinfection). Studies that also included patients who may have spontaneously eradicated HCV after infections may have underestimated the risk of LF progression and given less accurate estimates of the risk factors. Moreover, in this study we performed an extensive risk assessment evaluating in the same cohort both the impact of LF on clinical outcomes and predictors of LF with the objective to suggest possible causal networks and interventions targeted to factors that come first in the pathway of events. Moreover, to provide clinicians with an overall picture of risk factors, we have performed multivariate analyses for each outcome.

In conclusion, patients with advanced viro-immunological status, with identification as intravenous drug users and with high γGT are most-at-risk for LF progression and clinical complications of HIV disease, especially if heavily exposed to the NRTI class of antiretroviral drugs. Importantly, FIB-4 evaluated at baseline and during therapy is an independent predictor of these clinical complications. The complex network of LF and clinical evaluation in patients coinfected by HIV and HCV should be taken into account as they should provoke further pathogenetic and clinical investigations for optimal clinical management. In particular, test-and-treat strategies (leading to both earlier cART and earlier treatment for HCV) should be urgently implemented.

## Acknowledgments

The Italian MASTER Cohort is a large national project involving the major centers providing care to HIV/AIDS patients and includes the following doctors: Francesco Castelli, Eugenia Quiros-Roldan, Paola Nasta (Malattie Infettive Università degli Studi di Brescia); Alfredo Scalzini, Filippo Castelnuovo (Malattie Infettive Spedali Civili di Brescia); Elena Raffetti, Francesco Donato (Unità di Igiene, Epidemiologia e Sanità Pubblica, Università degli Studi di Brescia); Franco Maggiolo (Malattie Infettive Ospedale Papa Giovanni XXIII, Bergamo); Gioacchino Angarano, Nicoletta Ladisa (Clinica di Malattie Infettive Policlinico di Bari); Francesco Mazzotta, Massimo Di Pietro (Malattie Infettive S.M. Annunziata, Firenze); Andrea Gori, Giuseppe Lapadula, Silvia Costarelli (Malattie Infettive Ospedale San Gerardo di Monza); Laura Sighinolfi (Malattie Infettive Nuovo Polo Ospedaliero di Cona, Ferrara); Angelo Pan, Silvia Lorenzotti (Malattie Infettive Istituti Ospitalieri Cremona); Roberto Cauda, Simona Di Giambenedetto (Malattie Infettive Policlinico A. Gemelli—Università Cattolica di Roma); Carlo Torti, Maria Concetta Postorino (Università *Magna Graecia* di Catanzaro); Mattia Prosperi (University of Florida, USA); Nicola Mazzini (M.I.S.I. Foundation).

## Supplementary Material

Supplemental Digital Content
