# Supplementary material for: Liver fibrosis progression and clinical outcomes are intertwined: role of CD4+ T-cell count and NRTI exposure from a large cohort of HIV/HCV-coinfected patients with detectable HCV-RNA: A MASTER cohort study
Source: Medicine (Baltimore). 2016 Jul 22;95(29):e4091. doi: 10.1097/MD.0000000000004091 (PMC5265753; doi:10.1097/MD.0000000000004091)
Supplement: Supplemental Digital Content [file medi-95-e4091-s001.docx]

**Supplementary file:**

**Table 1:** Predictors of death and clinical events (composite outcome) and liver fibrosis progression (multivariable Cox’s regression analysis)

| Composite outcome (death and clinical events) | HR | 95%CI | | p-value |
| --- | --- | --- | --- | --- |
| year of first ART start | 1.01 | 0.99 | 1.03 | 0.1636 |
| non-idu vs. idu | 0.84 | 0.70 | 1.00 | 0.0510 |
| GGT per log higher* | 1.16 | 1.08 | 1.26 | **0.0001** |
| CD4+ per square root increase* | 0.96 | 0.95 | 0.97 | **<0.0001** |
| HIV RNA per log higher* | 0.96 | 0.90 | 1.02 | 0.1636 |
| HCV RNA per log higher* | 1.01 | 0.96 | 1.06 | 0.8011 |
| FIB-4 class2 vs. class1* | 0.99 | 0.84 | 1.17 | 0.9139 |
| FIB-4 class3 vs. class1* | 1.64 | 1.23 | 2.19 | **0.0008** |
| NRTI cumulative years* | 0.98 | 0.93 | 1.02 | 0.3405 |
| NNRTI cumulative years* | 0.92 | 0.86 | 0.97 | **0.0043** |
| PI cumulative years* | 1.00 | 0.96 | 1.05 | 0.8661 |
| RTV booster cumulative years* | 0.85 | 0.77 | 0.93 | **0.0004** |
| other ARVs cumulative years* | 0.86 | 0.62 | 1.18 | 0.3439 |
| male vs. female | 0.96 | 0.81 | 1.14 | 0.6595 |
| FIB-4 transition from class 1 to class 2 or 3 | **HR** | **95%CI** | | **p-value** |
| year of first ART start | 1.13 | 1.11 | 1.14 | **<0.0001** |
| non-idu vs. idu | 0.79 | 0.69 | 0.92 | **0.0016** |
| GGT per log higher* | 1.51 | 1.42 | 1.60 | **<0.0001** |
| CD4+ per square root increase* | 0.98 | 0.97 | 0.99 | **0.0003** |
| HIV RNA per log higher* | 1.37 | 1.30 | 1.44 | **<0.0001** |
| HCV RNA per log higher* | 1.01 | 0.98 | 1.05 | 0.4644 |
| FIB-4 baseline | 4.04 | 3.23 | 5.05 | **<0.0001** |
| NRTI cumulative years* | 1.15 | 1.09 | 1.21 | **<0.0001** |
| NNRTI cumulative years* | 0.95 | 0.91 | 0.99 | **0.0110** |
| PI cumulative years* | 1.00 | 0.96 | 1.04 | 0.9776 |
| RTV booster cumulative years* | 0.97 | 0.91 | 1.04 | 0.4017 |
| other ARVs cumulative years* | 0.91 | 0.73 | 1.14 | 0.4104 |
| AIDS event* | 1.19 | 0.97 | 1.45 | 0.0904 |
| Neurological event* | 1.08 | 0.70 | 1.67 | 0.7227 |
| Diabetes* | 0.47 | 0.19 | 1.17 | 0.1051 |
| Cardiovascular event* | 1.21 | 0.81 | 1.82 | 0.3595 |
| Kidney event* | 1.25 | 0.93 | 1.68 | 0.1335 |
| male vs. female | 0.94 | 0.81 | 1.08 | 0.3657 |
| FIB-4 transition from class 2 to class 3 | **HR** | **95%CI** | | **p-value** |
| year of first ART start | 1.12 | 1.09 | 1.15 | **<0.0001** |
| non-idu vs. idu | 0.76 | 0.58 | 1.00 | **0.0460** |
| GGT per log higher* | 1.76 | 1.57 | 1.96 | **<0.0001** |
| CD4+ per square root increase* | 0.98 | 0.96 | 1.00 | **0.0355** |
| HIV RNA per log higher* | 1.42 | 1.30 | 1.54 | **<0.0001** |
| HCV RNA per log higher* | 1.01 | 0.95 | 1.08 | 0.6612 |
| FIB-4 baseline | 1.83 | 1.50 | 2.23 | **<0.0001** |
| NRTI cumulative years* | 1.10 | 1.03 | 1.18 | **0.0077** |
| NNRTI cumulative years* | 0.97 | 0.90 | 1.04 | 0.4063 |
| PI cumulative years* | 1.06 | 0.99 | 1.13 | 0.0861 |
| RTV booster cumulative years* | 0.94 | 0.86 | 1.03 | 0.2187 |
| other ARVs cumulative years* | 1.13 | 0.84 | 1.52 | 0.4208 |
| AIDS event* | 1.16 | 0.85 | 1.58 | 0.3472 |
| Neurological event* | 1.25 | 0.73 | 2.14 | 0.4191 |
| Diabetes* | 1.92 | 0.60 | 6.13 | 0.2686 |
| Cardiovascular event* | 1.52 | 0.95 | 2.45 | 0.0818 |
| Kidney event* | 1.82 | 1.09 | 3.03 | **0.0221** |
| male vs. female | 0.82 | 0.63 | 1.07 | 0.1405 |
| FIB-4 increase of half a point from baseline | **HR** | **95%CI** | | **p-value** |
| year of first ART start | 1.12 | 1.11 | 1.13 | **<0.0001** |
| non-idu vs. idu | 0.82 | 0.73 | 0.92 | **0.0011** |
| GGT per log higher* | 1.51 | 1.44 | 1.59 | **<0.0001** |
| CD4+ per square root increase* | 0.98 | 0.97 | 0.99 | **<0.0001** |
| HIV RNA per log higher* | 1.36 | 1.31 | 1.42 | **<0.0001** |
| HCV RNA per log higher* | 1.01 | 0.98 | 1.04 | 0.3910 |
| FIB-4 baseline | 0.77 | 0.71 | 0.83 | **<0.0001** |
| NRTI cumulative years* | 1.12 | 1.08 | 1.16 | **<0.0001** |
| NNRTI cumulative years* | 0.98 | 0.94 | 1.01 | 0.1725 |
| PI cumulative years* | 1.04 | 1.00 | 1.07 | **0.0396** |
| RTV booster cumulative years* | 0.95 | 0.90 | 0.99 | **0.0301** |
| other ARVs cumulative years* | 0.91 | 0.76 | 1.10 | 0.3349 |
| AIDS event* | 1.39 | 1.19 | 1.61 | **<0.0001** |
| Neurological event* | 0.96 | 0.69 | 1.33 | 0.7920 |
| Diabetes* | 0.90 | 0.46 | 1.77 | 0.7649 |
| Cardiovascular event* | 1.23 | 0.91 | 1.66 | 0.1823 |
| Kidney event* | 1.35 | 1.06 | 1.72 | **0.0156** |
| male vs. female | 0.93 | 0.83 | 1.04 | 0.2202 |

* time-updated variables
